# Supplementary figures and images for: RPW8/HR repeats control NLR activation in Arabidopsis thaliana
Source: PLoS Genet. 2019 Jul 25;15(7):e1008313. doi: 10.1371/journal.pgen.1008313 (PMC6684095; doi:10.1371/journal.pgen.1008313)

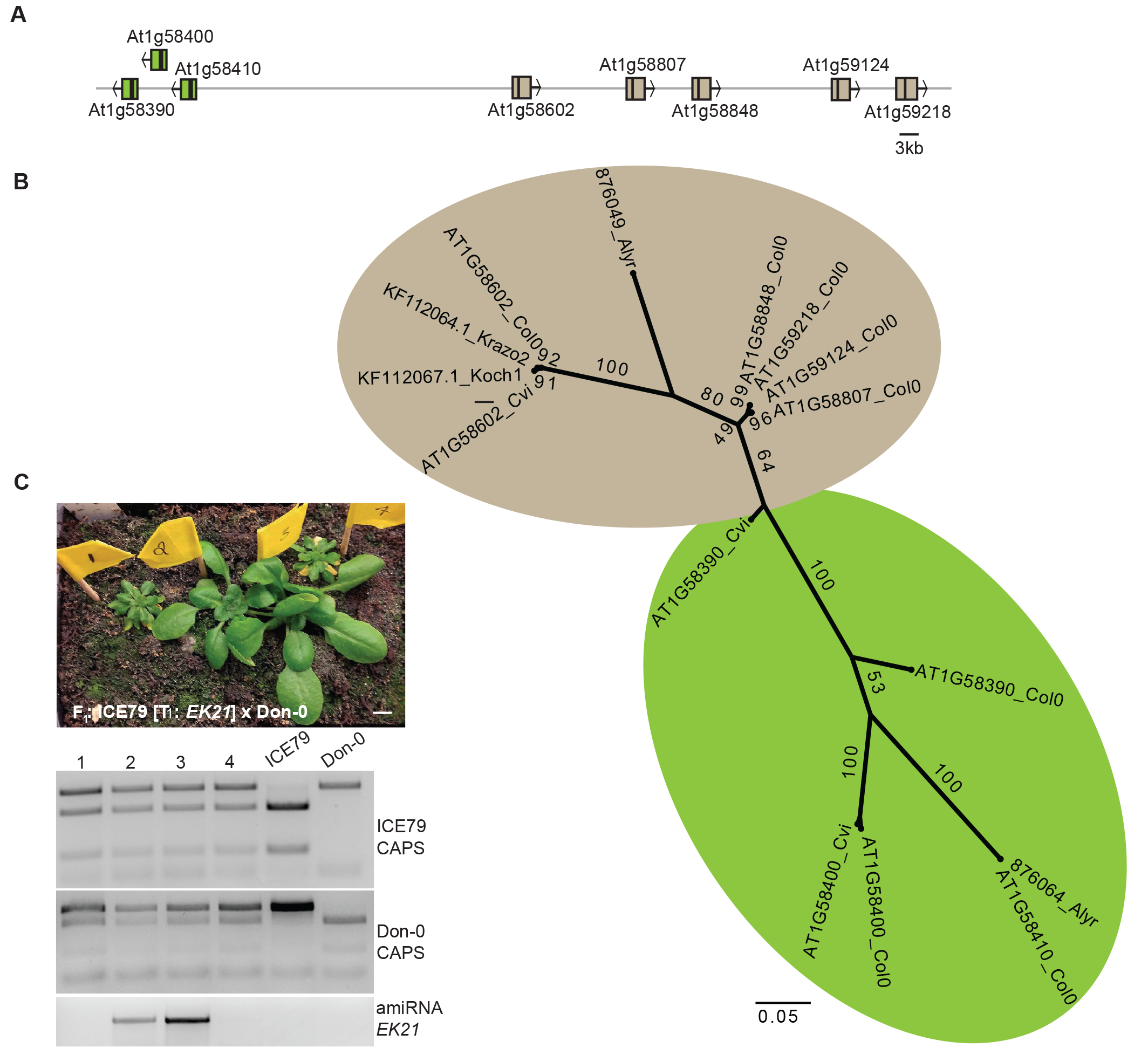

Supplement: S1 Fig — Related to Fig 1. (A) RPP7 cluster in the Col-0 reference genome. The left portion of the cluster consists of three NLR genes, At1g58390, At1g58400 and At1g58410 (green arrows). The right portion includes five NLR genes, At1g58602, At1g58807, At1g58848, At1g59124 and At1g59218 (brown arrows). Twenty-two non-NLR genes in this region are not shown. (B) Maximum-likelihood tree of NLR genes in the RPP7 cluster based on the NB domain. At1g59124 and At1g58807 sequences are identical, as are At1g59218 and At1g58848. Same colors as in (A). Bootstrap values (out of 100) are indicated on each branch. (C) Representative rescue experiment using an amiRNA construct targeting RPP7 homologs (see S1 Table). ICE79 was transformed with the amiRNA construct EK21 and T1 plants were crossed to Don-0, resulting in rescued and non-rescued plants segregating in the F1 progeny. Parental genotypes were confirmed with CAPS markers, shown below. Five-week old plants grown in 16°C are shown. (TIF) [file pgen.1008313.s001.tif]

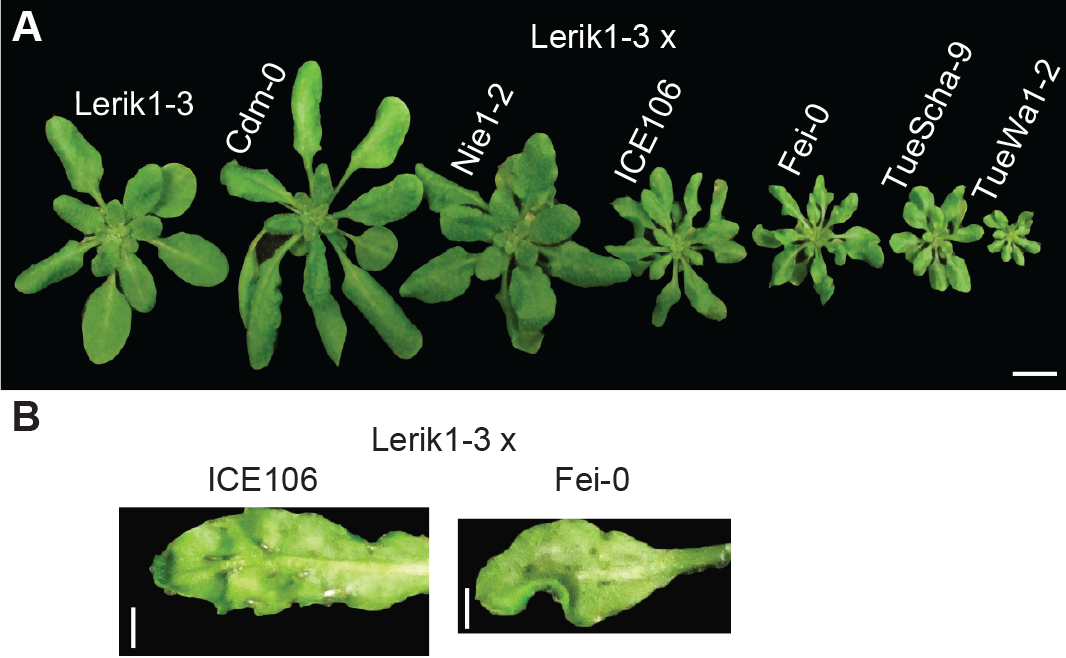

Supplement: S2 Fig — Related to Fig 1. Major differences were observed in rosette size of F1 hybrids (A) and spotted cell death on the abaxial side of leaves (B). Scale bar represents 1cm (A) and 1mm (B). Plants were five weeks old. (TIF) [file pgen.1008313.s002.tif]

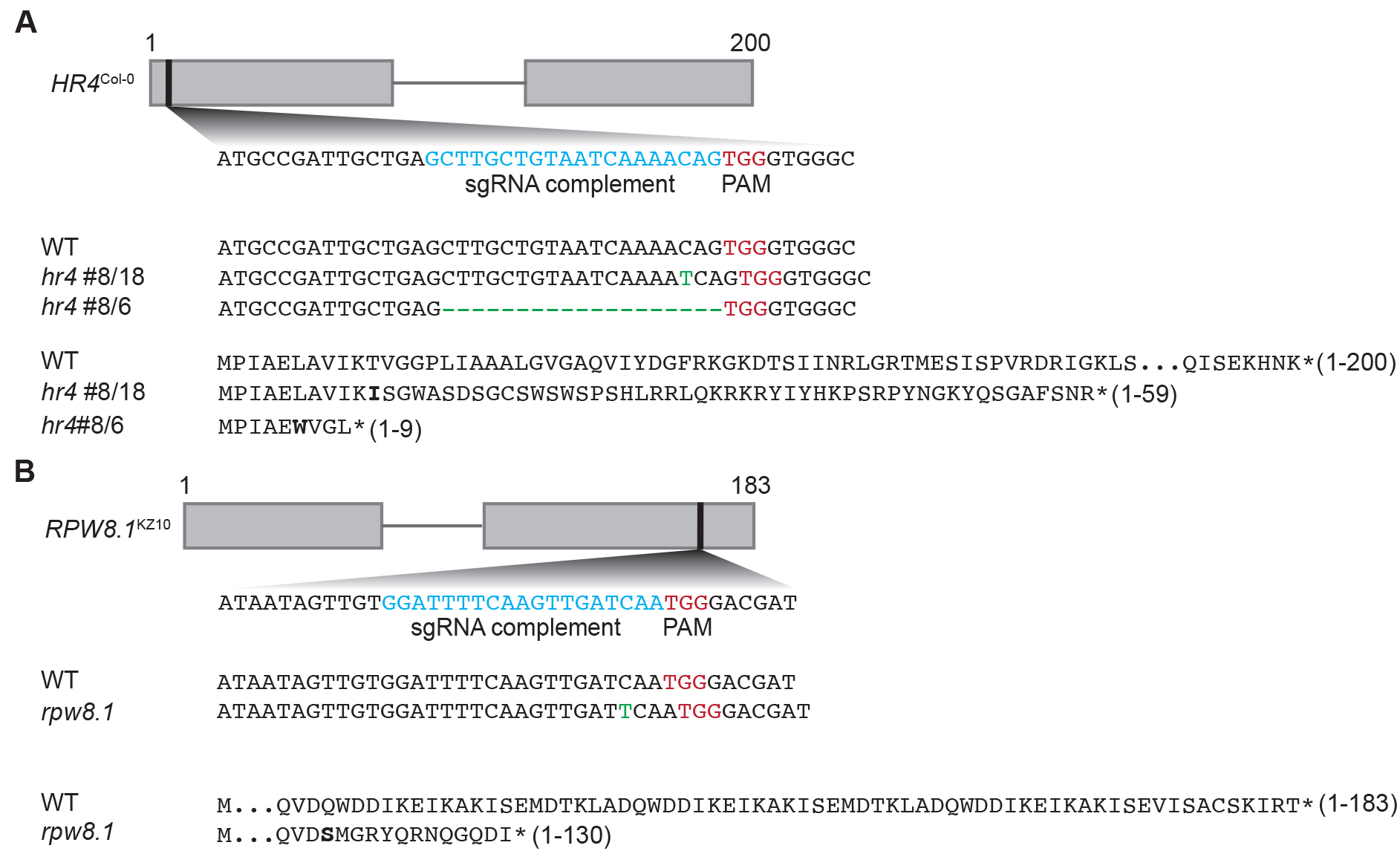

Supplement: S3 Fig — Related to Fig 3 and S4 Fig. (A) Two alleles of HR4 in Col-0 with a 1-bp insertion (#8/18) or a 19-bp deletion (#8/6) were identified by amplicon sequencing. (B) An allele of RPW8.1 in KZ10 with a 1-bp insertion was recovered. The stop codons are marked with an asterisk and the first amino acid after a frameshifting event is in bold. (TIF) [file pgen.1008313.s003.tif]

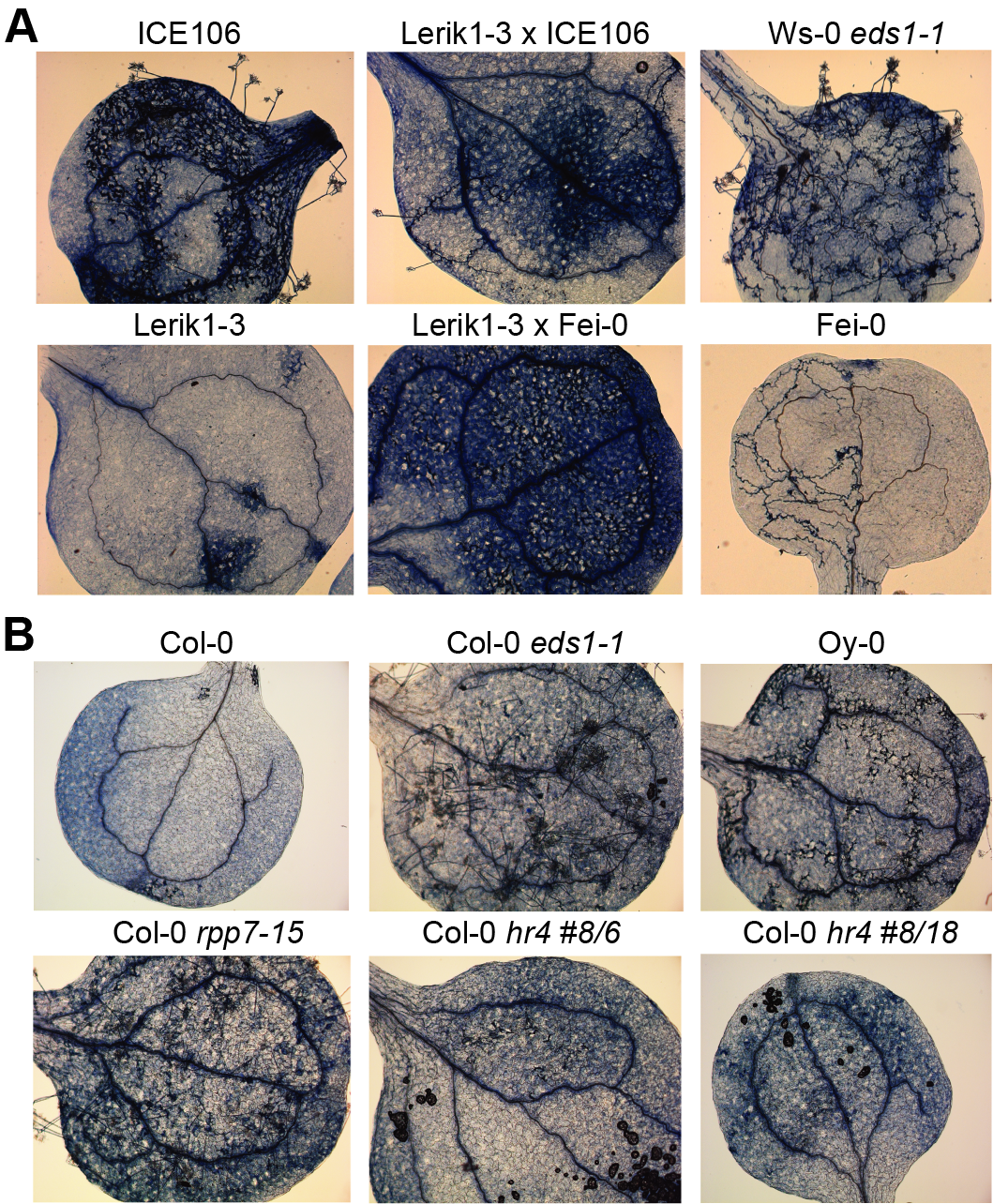

Supplement: S4 Fig — (A) Trypan Blue stained cotyledons 5 days after infection. Lerik1-3 is resistant, while Fei-0 and ICE106 are fully susceptible. The F1 hybrids Lerik1-3 x Fei-0 and Lerik1-3 x ICE106 appear to be less resistant than Lerik1-3. Ws-0 eds1-1 is a positive infection control. (B) Two different hr4 loss-of-function alleles (see S3 Fig) are as resistant as Col-0 wild-type plants. eds1-1 and rpp7-15 are positive infection controls. (TIF) [file pgen.1008313.s004.tif]

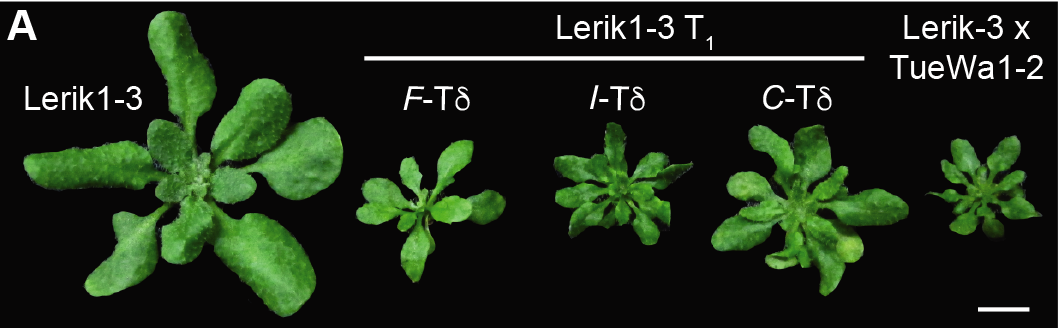

Supplement: S5 Fig — Related to Fig 5. Effects of chimeric HR4 transgenes introduced into Lerik1-3, with negative and positive controls shown to the left and right. Scale bar represents 1cm. Five week-old plants are shown. (TIF) [file pgen.1008313.s005.tif]

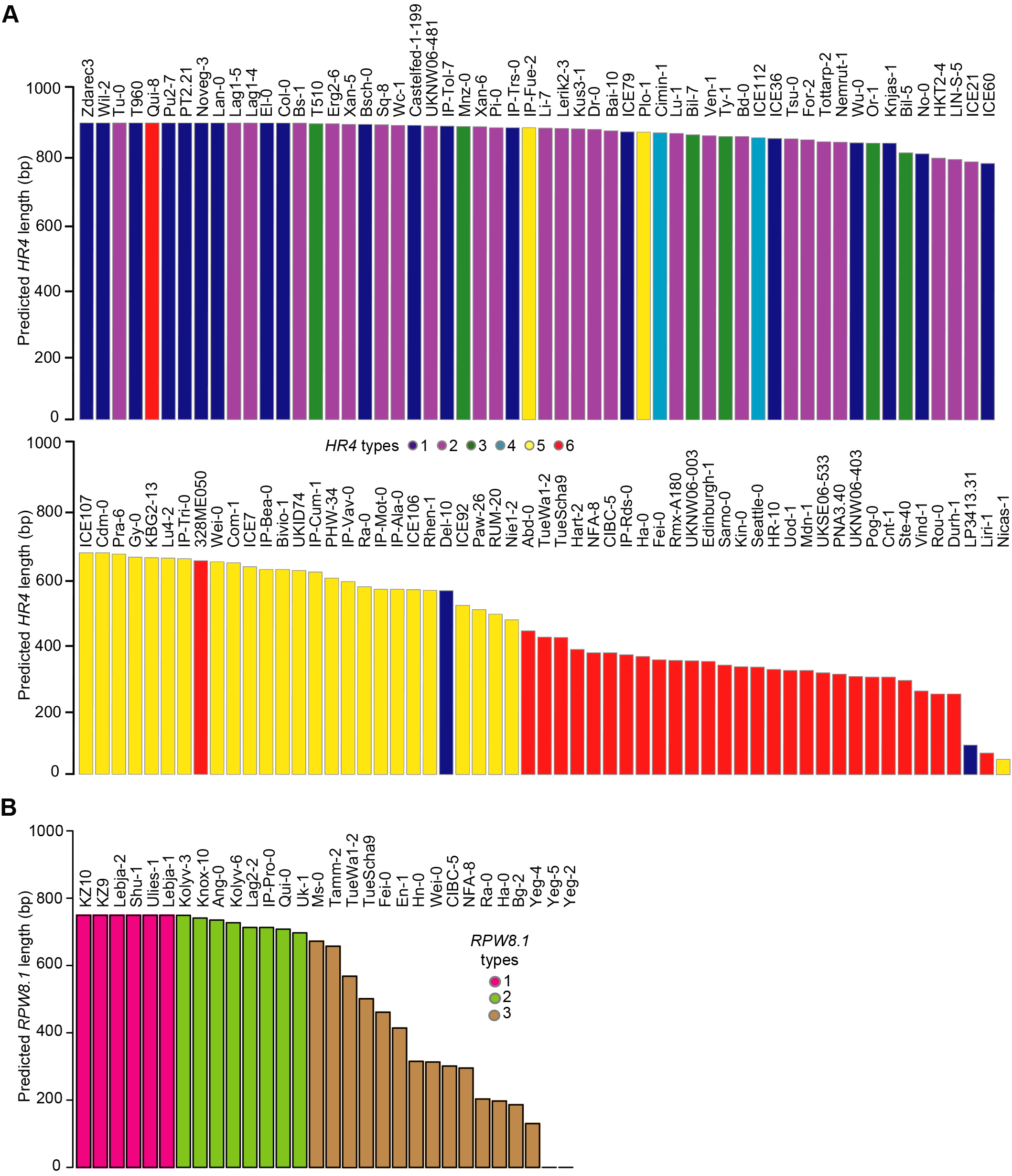

Supplement: S6 Fig — Related to Fig 6. (A) HR4 type assignments based on information from Sanger sequencing. (B) RPW8.1 type based on information from Sanger sequencing. (TIF) [file pgen.1008313.s006.tif]

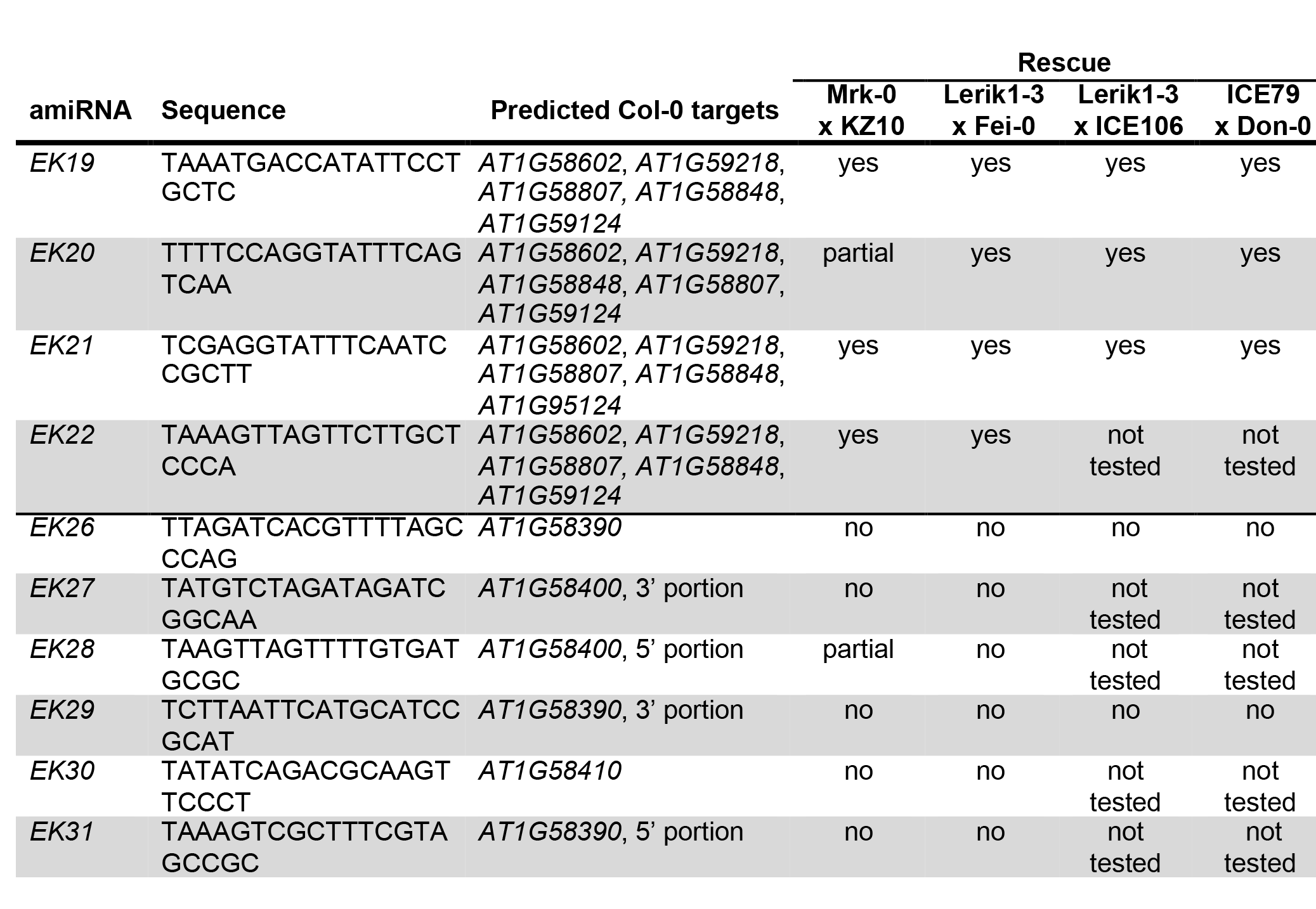

Supplement: S1 Table — Related to Fig 1. AmiRNAs were designed based on NLR sequences of the RPP7 cluster in Col-0 (Table S1) using WMD3 (http://wmd3.weigelworld.org/). Constructs were introduced into Mrk-0, Lerik1-3 or ICE79, and T1 lines were crossed to incompatible parents. Hybrid necrosis was scored at 16°C. Examples of F1 plants are shown in S1 Fig. (TIF) [file pgen.1008313.s007.tif]

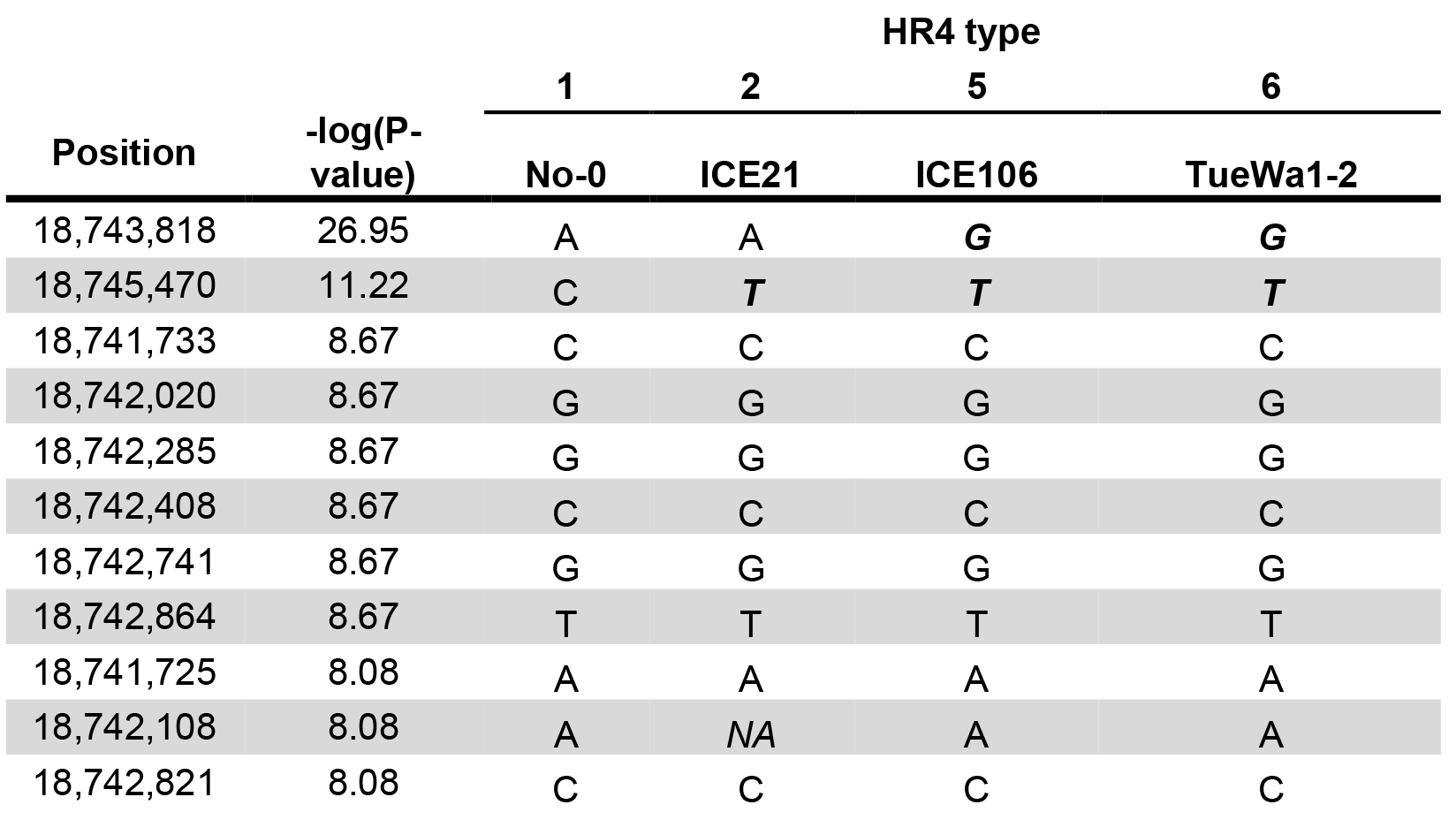

Supplement: S2 Table — Related to Fig 2. Location of HR4 (At3g50480) is 18,733,287 to 18,734,180 bp on chromosome 3 of the reference Col-0 genome. The next protein-coding gene is At3g50500 (18,741,805 to 18,743,904 bp), with At3g50490 (18,738,630 to 18,739,261 bp) encoding a transposable element (see Fig 4A). SNPs in bold italics differ from the Col-0 reference. (TIF) [file pgen.1008313.s008.tif]

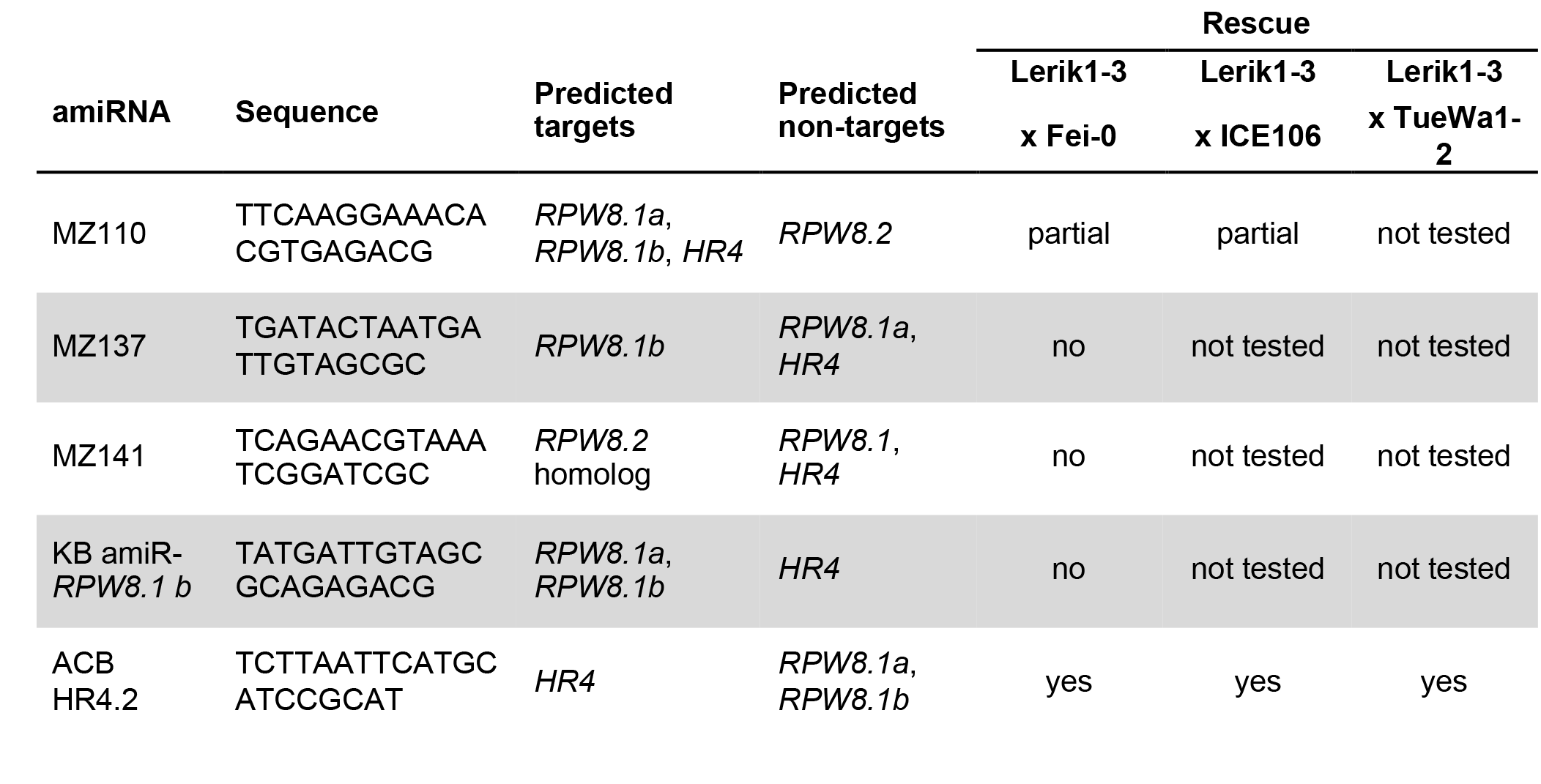

Supplement: S3 Table — Related to Figs 1 and 3. AmiRNAs were designed based on sequence information of RPW8/HR clusters from Col-0, Ms-0 and KZ10. Constructs were introduced into Fei-0 or ICE106, and T1 lines were crossed to the incompatible accession Lerik1-3. Hybrid necrosis was scored at 16°C. Parental genotypes and the presence of amiRNA constructs were confirmed by PCR genotyping (see Fig 3A). (TIF) [file pgen.1008313.s009.tif]

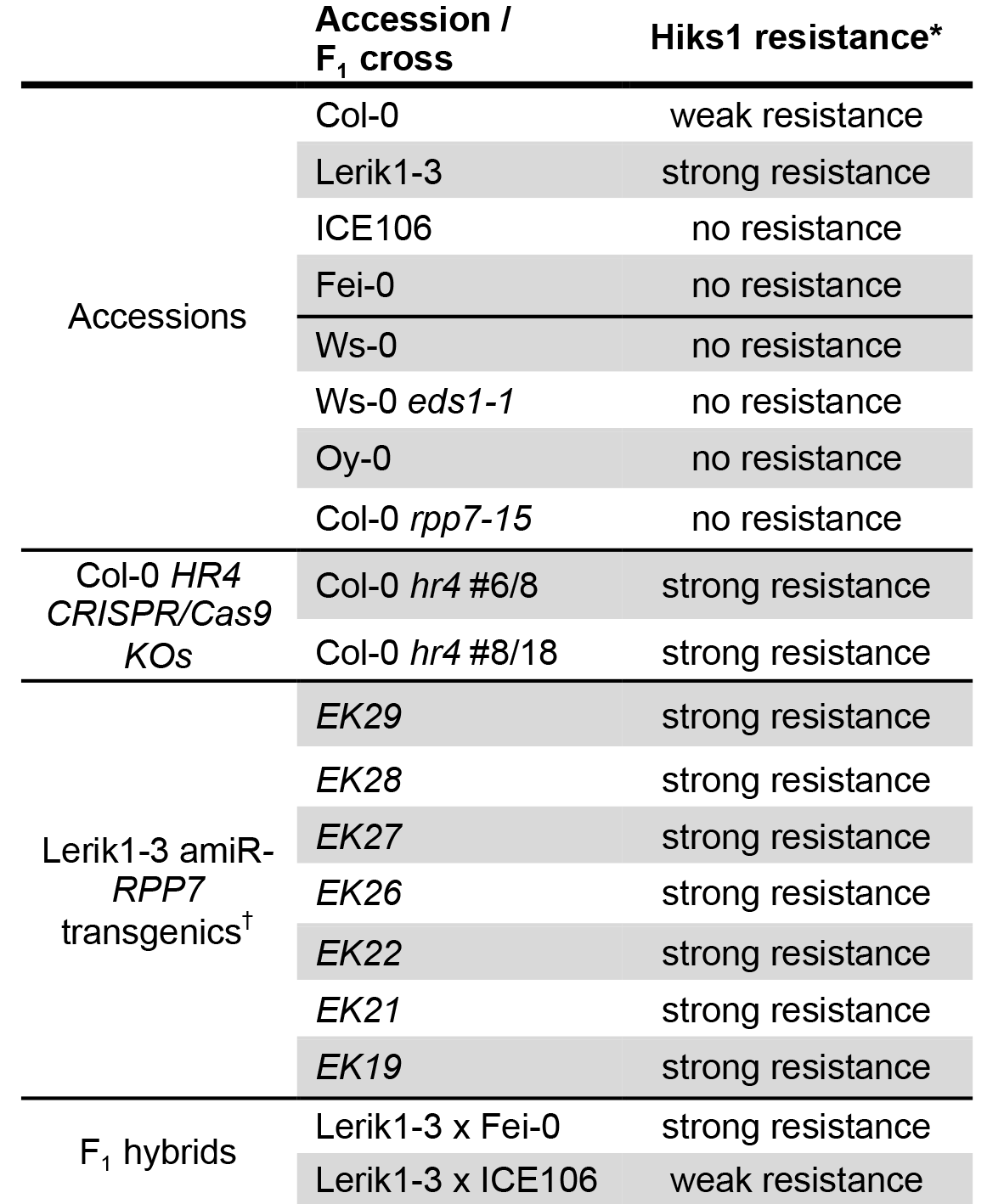

Supplement: S4 Table — Related to S4 Fig. *strong resistance: no conidiophores; weak resistance: 1–5 conidiophores/cotyledon, with some sporulation; very weak resistance: 6–19 conidiophores/cotyledon, with low to medium sporulation; no resistance: >20 conidiophores/cotyledon, heavy sporulation. †See S1 Table for amiRNA key. (TIF) [file pgen.1008313.s010.tif]

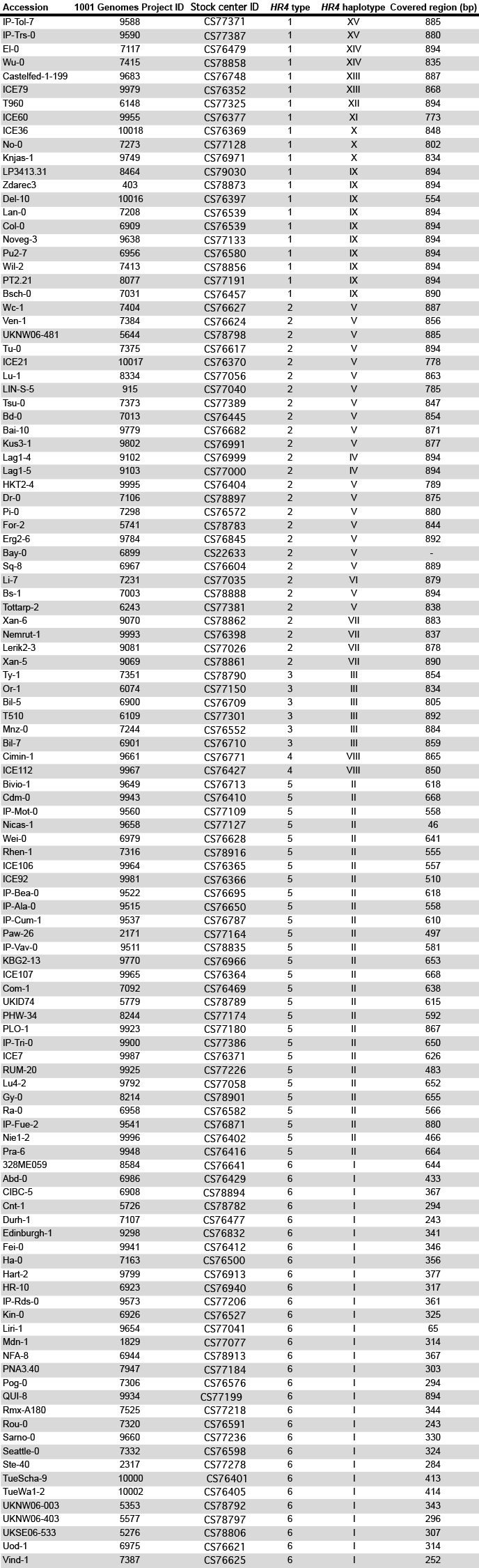

Supplement: S5 Table — Related to Fig 6. Covered region indicates the length of HR4Col-0 (894 bp) covered by reads from the 1001 Genomes Project (http://1001genomes.org), allowing for five mismatches. HR4 types are categorized according to the number of RPW8/HR repeats, and the haplotype is based on the entire HR4 coding sequence. (TIF) [file pgen.1008313.s011.tif]

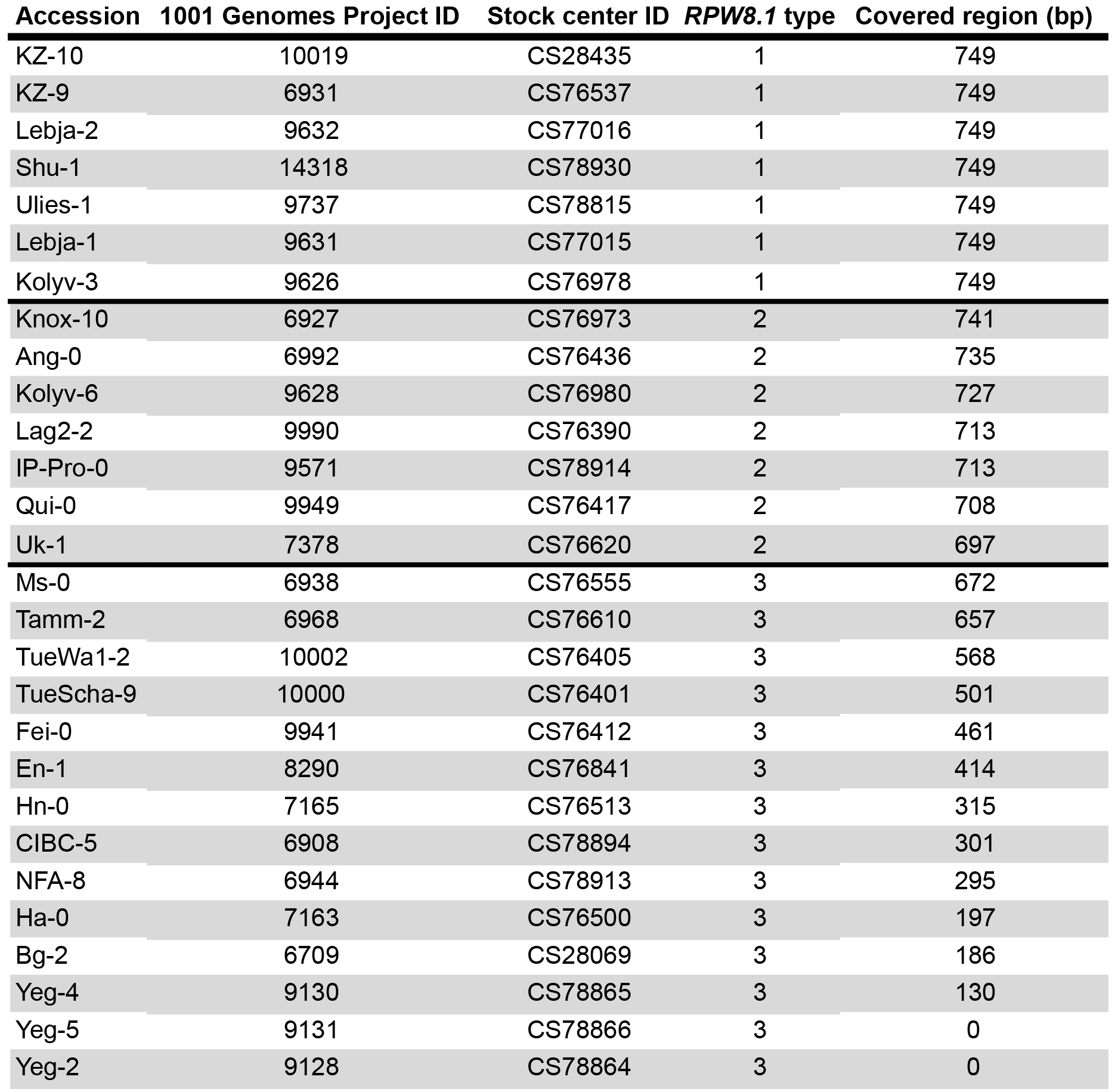

Supplement: S6 Table — Related to Fig 6. (TIF) [file pgen.1008313.s012.tif]

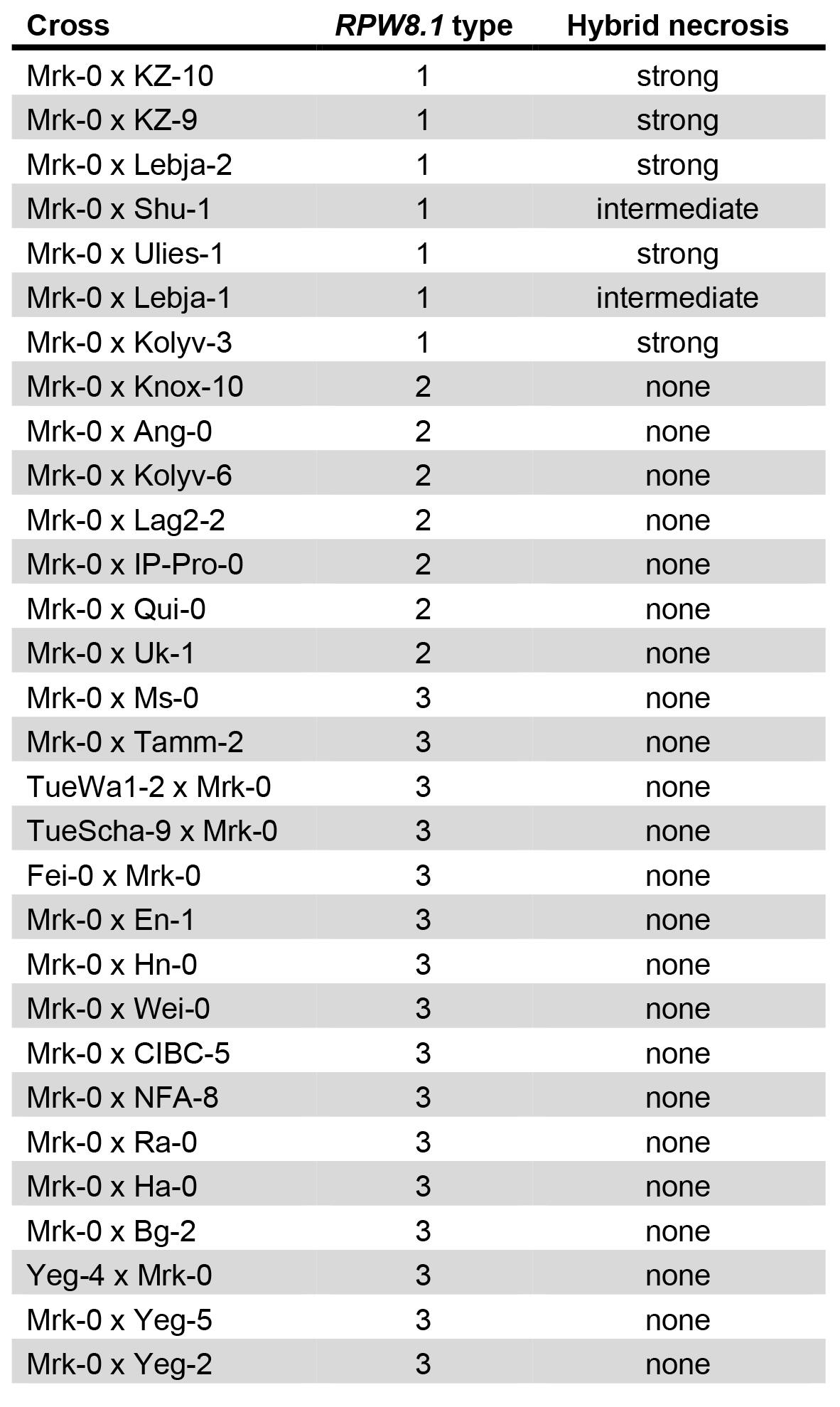

Supplement: S7 Table — Related to Fig 6. Strong hybrid necrosis equals what is observed in KZ10 x Mrk-0 hybrids. (TIF) [file pgen.1008313.s013.tif]

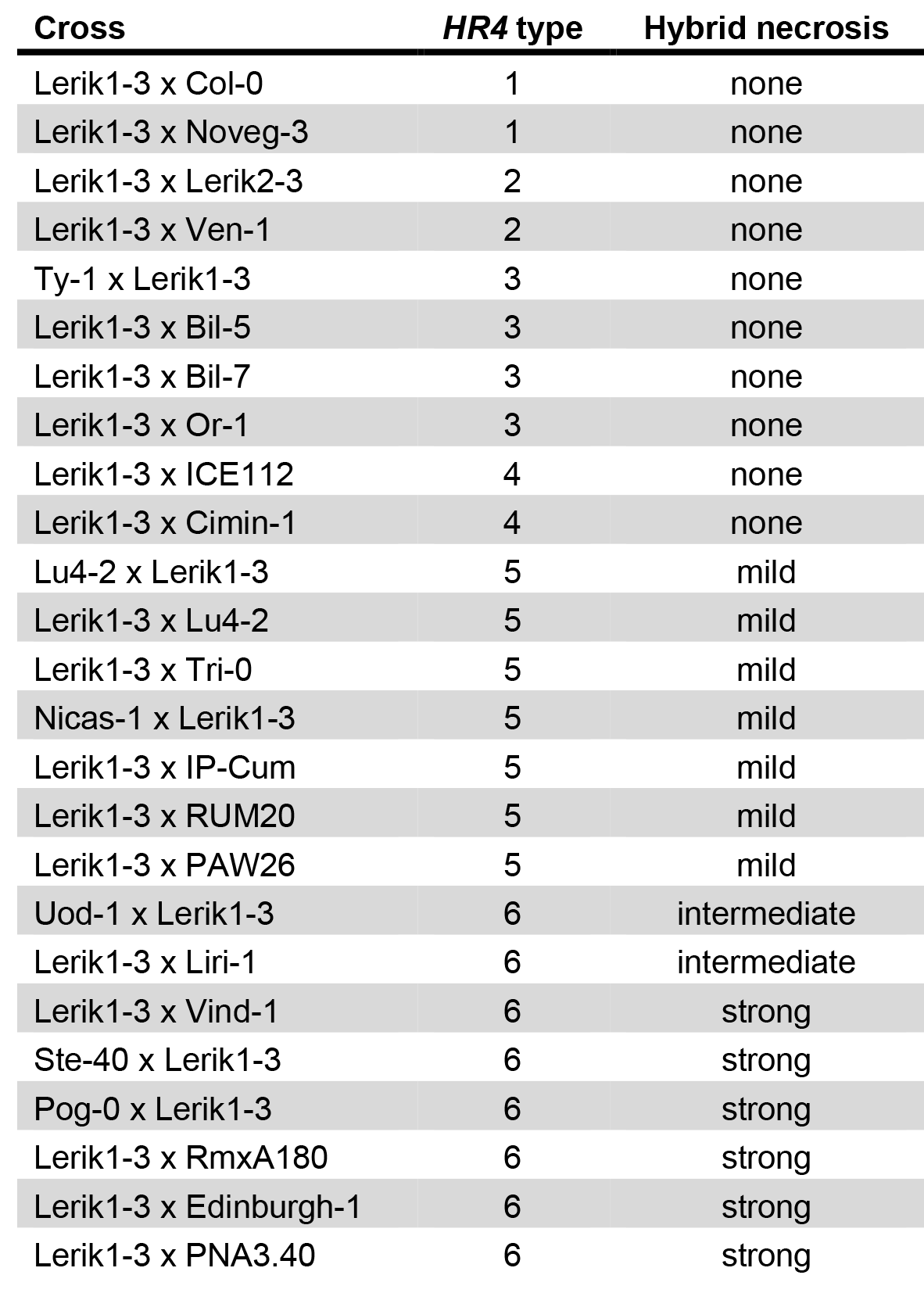

Supplement: S8 Table — Related to Fig 6. Strong hybrid necrosis equals what is observed in Lerik1-3 x Fei-0 F1 hybrids. (TIF) [file pgen.1008313.s014.tif]

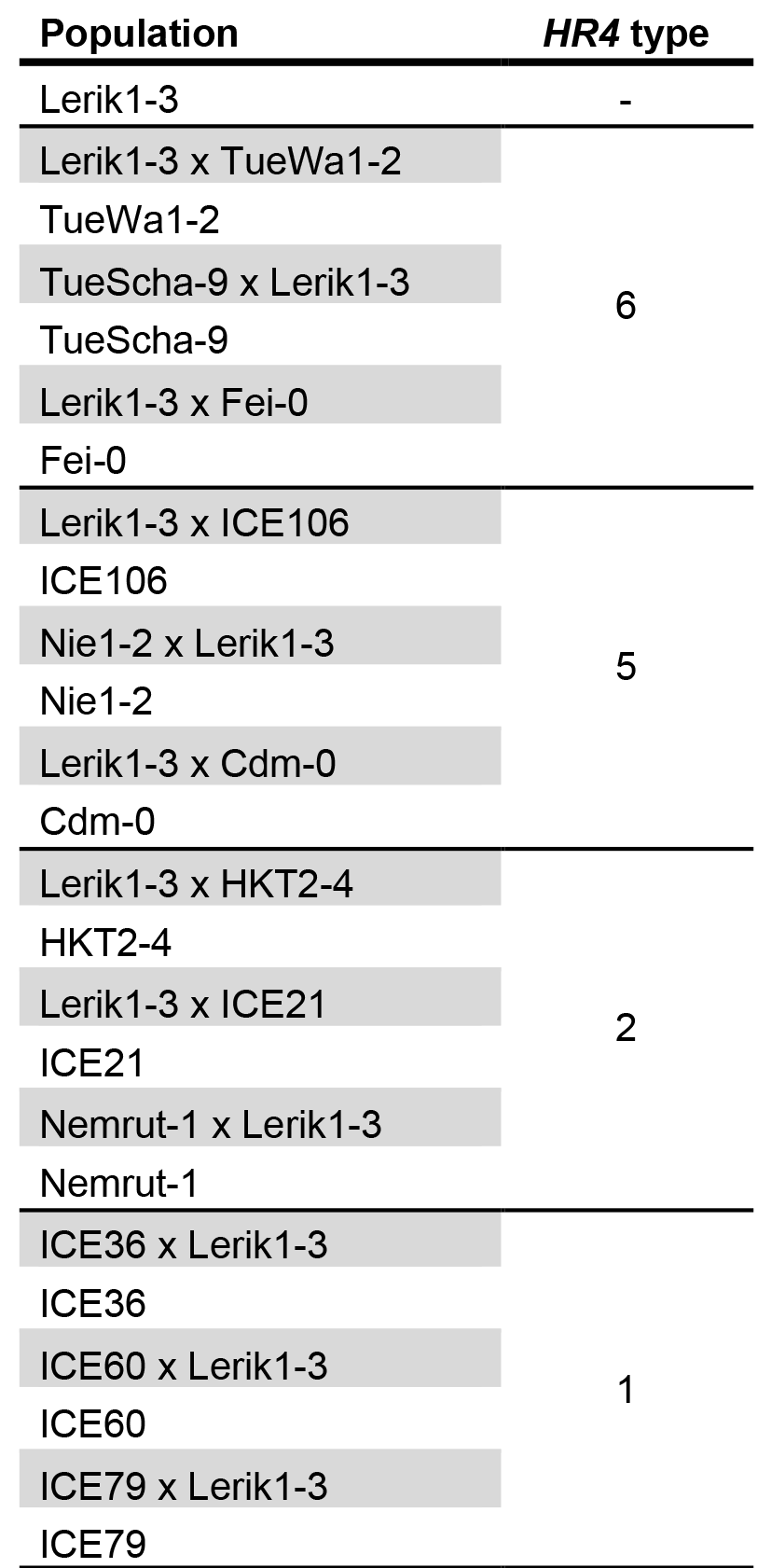

Supplement: S9 Table — Related to Fig 6. (TIF) [file pgen.1008313.s015.tif]

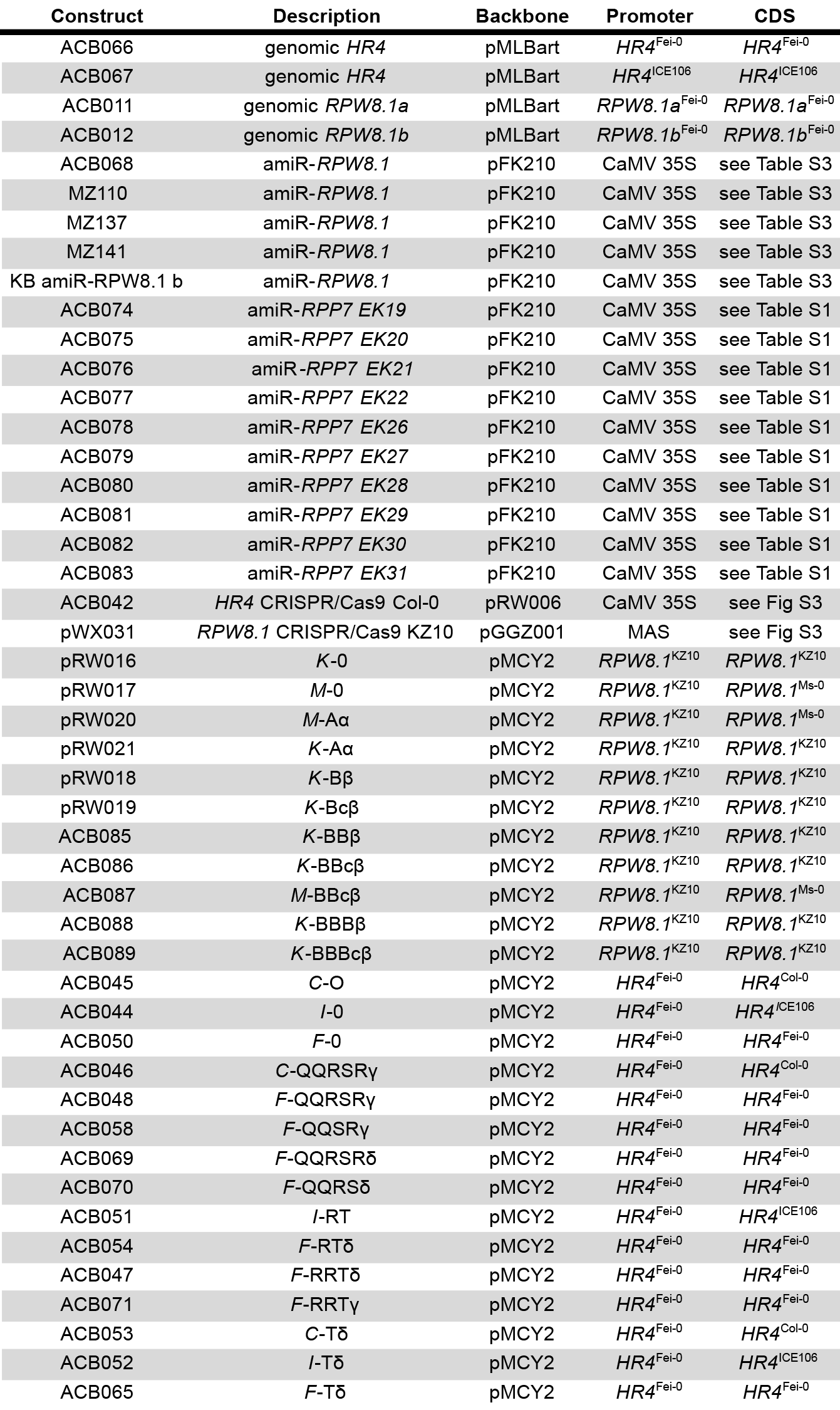

Supplement: S10 Table — (TIF) [file pgen.1008313.s016.tif]

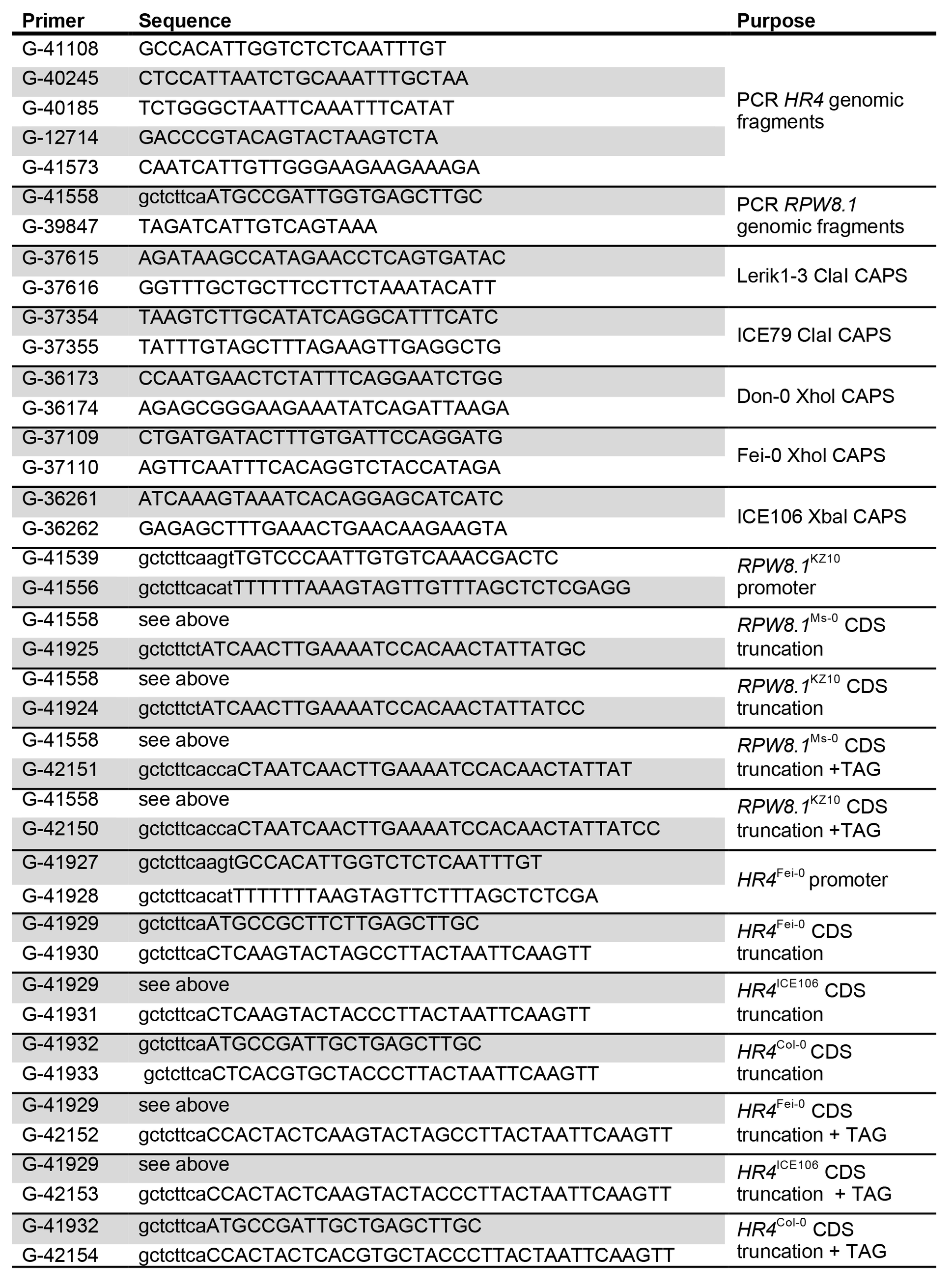

Supplement: S11 Table — Related to Figs 3 and 5. (TIF) [file pgen.1008313.s017.tif]
